# Quality Evaluation of Apocyni Veneti Folium from Different Habitats and Commercial Herbs Based on Simultaneous Determination of Multiple Bioactive Constituents Combined with Multivariate Statistical Analysis

**DOI:** 10.3390/molecules23030573

**Published:** 2018-03-03

**Authors:** Cuihua Chen, Zixiu Liu, Lisi Zou, Xunhong Liu, Chuan Chai, Hui Zhao, Ying Yan, Chengcheng Wang

**Affiliations:** College of Pharmacy, Nanjing University of Chinese Medicine, Nanjing 210023, China; cuihuachen2013@163.com (C.C.); liuzixiu3221@126.com (Z.L.); zlstcm@126.com (L.Z.); echo_0523@hotmail.com (C.C.); zhaohui_199301@163.com (H.Z.); yanying93ly@163.com (Y.Y.); ccw199192@163.com (C.W.)

**Keywords:** Apocyni Veneti Folium, multiple bioactive constituents, UFLC-QTRAP-MS/MS, simultaneous determination, multivariate statistical analysis

## Abstract

Apocyni Veneti Folium (AVF) is a kind of staple traditional Chinese medicine with vast clinical consumption because of its positive effects. However, due to the habitats and adulterants, its quality is uneven. To control the quality of this medicinal herb, in this study, the quality of AVF was evaluated based on simultaneous determination of multiple bioactive constituents combined with multivariate statistical analysis. A reliable method based on ultra-fast liquid chromatography tandem triple quadrupole mass spectrometry (UFLC-QTRAP-MS/MS) was developed for the simultaneous determination of a total of 43 constituents, including 15 flavonoids, 6 organic acids, 13 amino acids, and 9 nucleosides in 41 Luobumaye samples from different habitats and commercial herbs. Furthermore, according to the contents of these 43 constituents, principal component analysis (PCA) was employed to classify and distinguish between AVF and its adulterants, leaves of *Poacynum hendersonii* (PHF), and gray relational analysis (GRA) was performed to evaluate the quality of the samples. The proposed method was successfully applied to the comprehensive quality evaluation of AVF, and all results demonstrated that the quality of AVF was higher than the PHF. This study will provide comprehensive information necessary for the quality control of AVF.

## 1. Introduction

Apocyni Veneti Folium (AVF, Luobumaye in Chinese), derived from the dried leaves of *Apocynum venetum* L. (Apocynaceae), is one of the most popular traditional Chinese medicines (TCMs) and is officially documented in the Chinese Pharmacopoeia. It has been applied to treat cardiac disease, hypertension, nephritis, and neurasthenia for hundreds of years in China [1]. Modern studies in pharmacology and therapeutics have demonstrated that AVF has antihypertensive [2,3], antidepressant [4], hepatoprotective [5], anxiolytic [6], cardiotonic [7], antioxidative [8], and diuretic [9] effects. In Japan and Northern China, tea preparations from AVF have recently become popular because of their health benefits, specifically their ability to improve immunity and delay aging.

Phytochemical investigation has revealed that AVF contains several types of constituents, such as flavonoids, organic acids, amino acids, and nucleosides [10,11,12,13]. These constituents contained in AVF can exhibit various biological activities. For example, flavonoids and organic acids possess effective activities against inflammation, oxidation, cancer [14,15], viruses, and bacteria [16,17]; amino acids are important in human nutrition, affects palatability, and are involved in the regulation and modulation of various physiological processes in the body, including antiplatelet aggregation [18,19] and antihypertension [20]; nucleosides are bioactive substances that enhance immune activity, antiarrhythmic, antioxidant, and so on [21].

According to the Chinese Pharmacopoeia (2015 edition), the content of hyperoside in AVF has been employed as the quality evaluation index, which requires a concentration of no less than 0.3%. However, documents have shown that the content of single components may not be a rational basis for the quality control of AVF [22,23]. Most reports of quantitative analysis were concentrated on each particular class of the compounds or have focused on certain active ingredients for the quality control of AVF [24,25,26,27]. There is no published comparative study reported for the simultaneous quantification of flavonoids, organic acids, amino acids, and nucleosides in AVF, and the synergistic action of these various constituents is considered to be responsible for the therapeutic effects of TCM [28,29]. As a result, a comprehensive evaluation for quality control AVF requires a systematic method of simultaneously determining multiple bioactive constituents contained in this TCM.

At present, due to the shortage of AVF, the leaves of *Poacynum hendersonii* (Hook. f.) Woodson (PHF), a non-official plant species, have been used indiscriminately as a substitute and medicinal resource for many years regardless of their potential interspecific differences in components [30]. Adulteration with PHF seriously affects the safety and efficacy of AVF. Therefore, an effective method for distinguishing between the two species must be developed to ensure the quality of AVF.

The aim of our experiments is to evaluate the quality of AVF based on the simultaneous determination of multiple bioactive constituents combined with multivariate statistical analysis. A reliable method based on ultra-fast performance liquid chromatography coupled with triple quadrupole-linear ion trap mass spectrometry (UFLC-QTRAP-MS/MS) was established to simultaneously determine the content of 43 constituents including 15 flavonoids, 6 organic acids, 13 amino acids, and 9 nucleosides in 41 batches of Luobumaye samples from different habitats and commercial herbs. Furthermore, principal component analysis (PCA) was introduced to distinguish between AVF and its adulterant PHF, and gray relational analysis (GRA) was performed to evaluate the quality of the samples according to the contents of the tested constituents. The proposed method can be useful for the overall assessment on the quality of AVF.

## 2. Results and Discussion

### 2.1. Optimization of Sample Preparation

The extraction method (ultrasonic extraction and refluxing extraction), the extraction solvents (water, 25% methanol or ethanol, 50% methanol or ethanol, 75% methanol or ethanol, 100% methanol, and 95% ethanol), the solvent-to-sample ratios (12.5:1, 25:1, 50:1, 100:1, 200:1 (*v*/*w*)), and the extraction times (15 min, 30 min, 45 min, and 60 min) were optimized in order to obtain the appropriate extraction efficiency of four kinds of analytes: uracil, phenylalanine, neochlorogenic acid, and hyperoside/isoquercetin, respectively. Results showed that ultrasonic extraction with a 100:1 ratio of water for 45 min at room temperature were the conditions that allowed a higher yield of extraction of the analyzed compounds (Table S1).

### 2.2. Optimization of UFLC Conditions

Notably, aglycones have the property of hydrophobicity, and the glycosides are hydrophilic in flavonoids. Organic acids, amino acids, and nucleosides have high hydrophilicity. Hydrophilic chromatography columns, such as an Acquity UPLC BEH Amide (100 mm × 2.1 mm, 1.7 μm) or a Waters Atlantis T3 (100 mm × 2.1 mm, 5 μm) column, have extremely weak or no retention ability to polar-weak flavonoids. Consequently, taking into account the hydrophilic and hydrophobic radical group, C_18_ chromatography columns including a Thermo Acclaim^TM^ RSLC 120 C_18_ (150 mm × 2.1 mm, 2.2 μm) and an Agilent ZORBAX SB-C_18_ column (250 mm × 4.6 mm, 5 μm) were both compared to test samples. UFLC results showed that the latter was better than the former in separating effect. Different mobile phases (for example, acetonitrile/water, methanol/water, acetonitrile/0.1% aqueous formic acid, methanol/0.1% aqueous formic acid, acetonitrile containing 0.2% formic acid solution/0.2% aqueous formic acid, and methanol containing 0.2% formic acid solution/0.2% aqueous formic acid), flow rates (0.4 mL/min, 0.8 mL/min, and 1.0 mL/min) and column temperatures (25, 30, and 35 °C) were examined and compared. Finally, it was determined that a gradient elution using acetonitrile containing 0.2% formic acid as Eluent A and 0.2% formic acid solution as Eluent B at a flow rate of 1.0 mL/min under the column temperature of 30 °C resulted in the desired separation in a short analysis time.

### 2.3. Optimization of MS Conditions

**Figure 1:**
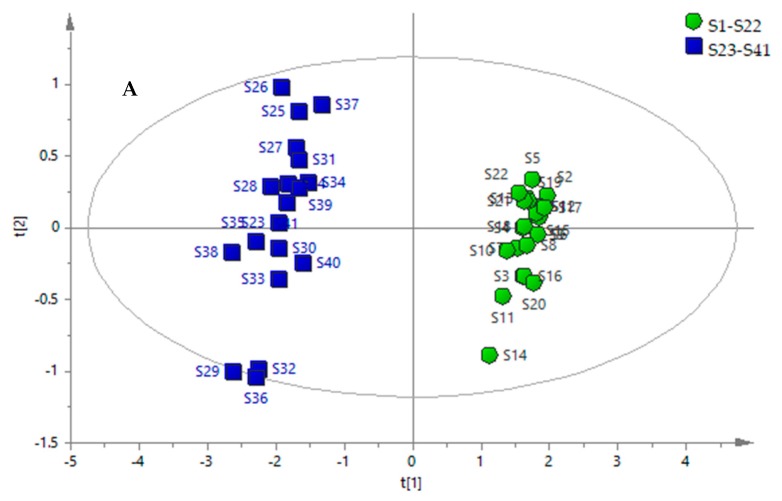

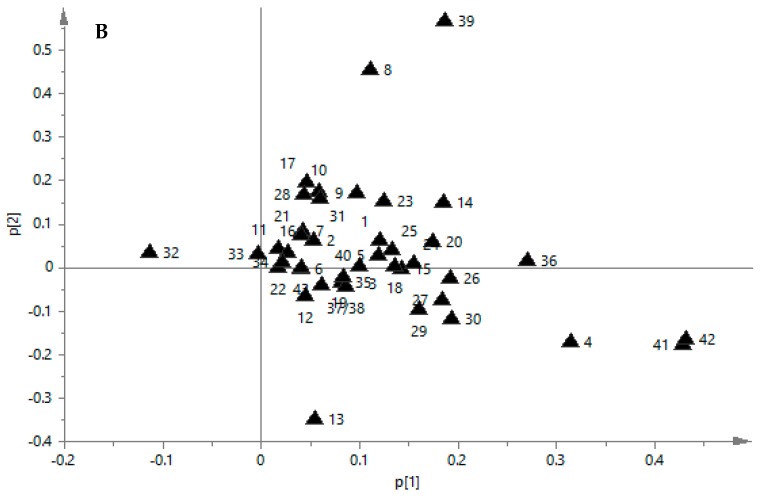
Multivariate statistical analysis of 41 samples of Apocyni Veneti Folium (AVF) and *Poacynum hendersonii* (PHF). Principal component analysis (PCA) scores plot (**A**) and PCA loading plot (**B**) of samples.

In order to obtain a sensitive and accurate quantitative method, individual solutions of all standard compounds (about 100 ng/mL) were injected separately into the electrospray ionization (ESI) source by a full-scan mass spectrometry (MS) method in direct infusion mode. Each compound was examined in both positive and negative modes to optimize the parameters of fragmentor voltage (FV) and collision energy (CE) with the highest sensitivity. After trial and error inspection, the vast majority of flavonoids and organic acids have a good condition in the negative ion mode. However, amino acids, nucleosides, and five flavonoids (trifolin, kaempferol-3-O-rutinoside, quercetin-3-O-sophoroside, avicularin, and amentoflavone) responded strongly in the positive ion mode. MRM (multiple reaction monitoring) transition from MS/MS spectrum was chosen when the most abundant, specific, and stable fragment ions appeared. Representative extract ion chromatograms of 43 analytes in MRM mode were presented in Figure 1, the detailed information of retention time (*t*_R_), MS information ([M − H]^−^ or [M + H]^+^), FV and CE for each analyte was listed in Table 1, and total ion chromatograms (TIC) of AVF and PHF are shown in Figure S1. As shown, catechin-epicatechin, gallocatechin-epigallocatechin, chlorogenic acid-neochlorogenic acid-cryptochlorogenic acid, hyperoside-isoquercetin, and leucine-isoleucine were isomers with the same precursor ion-product ion pairs; therefore, single reference substance was injected to UFLC-QTRAP-MS/MS to determine the compound with the help of *t*_R_. As hyperoside had the same *t*_R_ and precursor ion-product ion pairs with isoquercitrin and both of them were main flavonoids in AVF [22,23], these two analytes were identified as hyperoside/isoquercitrin together. Under the present condition of chromatographic and MS, constituents were identified and calculated by searching the database and the literature.

### 2.4. Method Validation

All method validations for quantification were performed using the UFLC-QTRAP-MS/MS technique. The calibration curve was obtained by injecting each analyte three times and more than six appropriate concentrations. The data are summarized in Table 2. The determination coefficients (*r*^2^ > 0.9990) indicate that all calibration curves had good linearity within the test ranges. The limits of detection (LODs) and limits of quantitation (LOQs) were measured in the range of 0.31–41.14 ng/mL and 1.02–137.13 ng/mL, respectively. The intra-day, inter-day, repeatability, stability test of the 43 analytes are presented with an RSD less than 5%, and the overall recoveries lay between 95.12% and 104.3%, with an RSD of no more than 4.76%. The approach of a calibration curve using standard samples with known analyte concentration was built to account for matrix effects. The slope ratio values of the matrix curve to pure solution curve are between 0.95 and 1.06, indicating that the matrix effect on the ionization of analytes was not obvious under optimized conditions. The results are shown in Table 2.

### 2.5. Sample Determination

Forty-three batches of samples from different habitats and commercial herbs are listed in Table 3. It can be seen that the origin of AVF was Tianjin, Xinjiang, Jilin, and Jiangsu, respectively, and the origin of PHF was Xinjiang, Tianjin, Guangxi, Hebei, and Nei Menggol, respectively. The developed UFLC-QTRAP-MS/MS method was subsequently applied to the comprehensive quality evaluation of samples of AVF and PHF. The results of the quantitative determination of the 43 analytes from these samples summarized in Table S2 indicated that significant differences were shown in bioactive constituents between these two species. AVF mainly contained hyperoside/isoquercitrin, quercetin-3-O-sophoroside, epicatechin, gallocatechin, chlorogenic acid, neochlorogenic acid, cryptochlorogenic acid, asparagine, and glutamine. The representative composition of AVF was hyperoside/isoquercitrin, with its content up to 5118.49 μg/g, much higher than that in PHF. While the main constituents of PHF were hyperoside/isoquercetin, quercetin-3-O-sophoroside (as high as 12,745.06 μg/g, about twice as much as that in AVF), epicatechin, epigallocatechin, and chlorogenic acid. Great differences were displayed between these two species in the contents of amenoflavone, chlorogenic acid, trifolin, neochlorogenic acid, astragalin, 2′-deoxyguanosine, glutamic acid, asparagine, isoleucine, and quercetin-3-O-sophoroside. Moreover, in addition to quercetin-3-O-sophoroside, the contents of other significant variation ingredients in AVF were much higher than that in PHF.

The total contents of flavonoids, organic acids, amino acids and nucleosides in AVF and PHF range from 25,473.72 to 21,385.70 μg/g and from 21,003.32 to 17,761.11 μg/g, respectively; 15 flavonoids range from 13,592.62 to 10,396.30 μg/g and from 17,111.8 to 14,053.08 μg/g, respectively; 6 organic acids range from 11,175.56 to 8486.02 μg/g and from 3097.35 to 1705.86 μg/g, respectively; 13 aminos acids range from 2444.02 to 1375.43 μg/g and from 994.09 to 414.47 μg/g, respectively; and 9 nucleosides range from 177.95 to 84.43 μg/g and from 129.52 to 29.90 μg/g, respectively. As can be seen in the above, significant differences were exhibited based on these bioactive constituents between AVF and PHF.

### 2.6. PCA of Samples

PCA based on the content of multiple bioactive constituents was used to classify and differentiate AVF and PHF. In the PCA scores plot (Figure 1A), each coordinate represents a sample, and it could be observed that the determined samples were clearly divided into two clusters: AVF was mostly located in a PC 1 positive axis; however, PHF was mostly distributed in a PC 1 negative axis. The first two principal components (PC 1 and PC 2) with >80% of the total variance were extracted for analysis. Among them, PC 1 and PC 2 accounted for 75.0% and 4.67% of the total variance, respectively. In the PCA loading plot (Figure 1B), the ions detected with large loading values can be considered as those that strongly contribute to the classification of those samples. The constituents of apigenin, amenoflavone, asparagine, trifolin, epigallocatechin, quercetin-3-O-sophoroside, and kaempferol-3-O-rutinoside were suggested to be diagnostic chemical markers for differentiating Luobumaye samples. As can be seen, PCA might be an effective method of evaluating the quality of AVF and PHF by virtue of the distribution on the PC axis.

### 2.7. GRA of Samples

GRA provides a reliable guarantee for the quality evaluation of traditional Chinese medicine. GRA was performed to evaluate the variation of AVF and PHF on the basis of the contents of 43 constituents. The gray comprehensive evaluation values (*r_i_*) and quality-rankings are listed in Table 4. Obviously, from a quality point of view, AVF is better than PHF, with *r_i_* values ranging from 0.5895 to 0.4653 and from 0.3780 to 0.2937, respectively, and there was no quality crossover between these two species. In general, the quality level of AVF from the main producing area of Tianjin was quite different, and the same was true for PHF from the main producing area of Xinjiang. The quality of PHF from Guangxi, compared to that from other regions, does not appear to be as high. Perhaps, these imply that geographical location and growth environment have a certain impact on the quality of AVF and PHF. In summary, the quality of Luobumaye samples can be successfully evaluated by GRA based on the contents of their multiple constituents. 

## 3. Materials and Methods

### 3.1. Chemicals and Reagents

Methanol and acetonitrile of HPLC grade were purchased from Merck (Damstadt, Germany). Ultrapure water was prepared using a Milli-Q purifying system (Millipore, Bedford, MA, USA). Other reagents were analytical grade (China National Pharmaceutical Group Co., Beijing, China). The chemical structures of 43 constituents, including glutamic acid (**1**), histidine (**2**), arginine (**3**), asparagine (**4**), serine (**5**), lysine (**6**), glutamine (**7**), cysteine (**8**), cytidine (**9**), guanine (**10**), 2′-deoxycytidine (**11**), uracil (**12**), hypoxanthine (**13**), tyrosine (**14**), isoleucine (**15**), guanosine (**16**), inosine (**17**), 2′-deoxyguanosine (**18**), fumaric acid (**19**), leucine (**20**), gallic acid (**21**), thymidine (**22**), phenylalanine (**23**), neochlorogenic acid (**24**), tryptophan (**25**), epigallocatechin (**26**), chlorogenic acid (**27**), catechin (**28**), cryptochlorogenic acid (**29**), caffeic acid (**30**), epicatechin (**31**), quercetin-3-O-sophoroside (**32**), quercitrin (**33**), avicularin (**34**), astragalin (**35**), trifolin (**36**), hyperoside (**37**), isoquercetin (**38**), kaempferol-3-O-rutinoside (**39**), rutin (**40**), amentoflavone (**41**), apigenin (**42**), and gallocatechin (**43**), are shown in Figure S3. The reference substances of **21**, **31**, **33**, **37**, and **40** were purchased from the Chinese National Institute of Control of Pharmaceutical and Biological Products (Beijing, China). The reference substances of **19**, **26**, **29**, **39**, **41**, **42**, and **43** were purchased from Chengdu Chroma Biotechnology (Chengdu, China). The reference substances of **1**–**18**, **20**, **22**, **23**, and **25** were purchased from Shanghai Yuanye Biotechnology (Shanghai, China). The reference substances of **24**, **27**, **28**, **30**, **32**, **34**, **35**, **36**, and **38** were purchased from Baoji Chenguang Biotechnology Co., Ltd. (Baoji, China). The purities of all of the above reference substances were greater than 98%, determined by HPLC analysis.

### 3.2. Plant Materials

Forty-one samples of AVF (S1–S22) and PHF (S23–S41) were obtained in different pharmacies or local Chinese herbal medicine market. Detailed information is shown in Table 3. The botanical origins of the materials were identified by Professor Xunhong Liu (Department for Authentication of Chinese Medicines, Nanjing University of Chinese Medicine, Nanjing, China), and the voucher specimens, named the same as those in Table 3, were deposited at the Herbarium in School of Pharmacy, Nanjing University of Chinese Medicine, China.

### 3.3. Preparation of Standard Solutions

A mixed standard stock solution containing 43 reference substances of **1**–**43** was prepared by dissolving them in water and their concentrations were as follows: (**1**) 5.02 μg/mL, (**2**) 3.22 μg/mL, (**3**) 3.86 μg/mL, (**4**) 21.37 μg/mL, (**5**) 5.72 μg/mL, (**6**) 4.58 μg/mL, (**7**) 12.31 μg/mL, (**8**) 3.31 μg/mL, (**9**) 7.00 μg/mL, (**10**) 2.57 μg/mL, (**11**) 2.38 μg/mL, (**12**) 0.60 μg/mL, (**13**) 0.65 μg/mL, (**14**) 3.05 μg/mL, (**15**) 7.92 μg/mL, (**16**) 0.58 μg/mL, (**17**) 4.04 μg/mL, (**18**) 6.94 μg/mL, (**19**) 8.88 μg/mL, (**20**) 5.68 μg/mL, (**21**) 1.49 μg/mL, (**22**) 1.75238 μg/mL, (**23**) 3.21 μg/mL, (**24**) 254.36 μg/mL, (**25**) 17.18 μg/mL, (**26**) 48.60 μg/mL, (**27**) 45.20 μg/mL, (**28**) 6.93 μg/mL, (**29**) 95.20 μg/mL, (**30**) 16.25 μg/mL, (**31**) 26.86 μg/mL, (**32**) 85.87 μg/mL, (**33**) 3.29 μg/mL, (**34**) 13.86 μg/mL, (**35**) 21.22 μg/mL, (**36**) 14.44 μg/mL, (**37**) 88.21 μg/mL, (**38**) 76.37 μg/mL, (**39**) 11.67 μg/mL, (**40**) 4.45 μg/mL, (**41**) 11.15 μg/mL, (**42**) 13.84 μg/mL, and (**43**) 18.08 μg/mL. The mixed standard stock solution was then diluted with water to a series of appropriate concentrations for construction of the calibration curves. All of the solutions were stored at 4 °C and filtered through 0.22 μm membranes (Jinteng laboratory equipment Co., Ltd., Tianjin, China) prior to injection for LC-MS analysis.

### 3.4. Preparation of Sample Solutions

The dried sample powders of AVF and PHF of about 0.5 g, after they were passed through a 40 mesh sieve, were weighed accurately, ultrasonically extracted with 50 mL of water for 45 min, cooled at room temperature, and then supplemented with water to compensate for the lost weight. The extract solution was subsequently centrifuged at 12,000 rpm for 10 min. The supernatant was stored at 4 °C and filtered through a 0.22 μm membrane (Jinteng laboratory equipment Co., Ltd., Tianjin, China) prior to analysis.

### 3.5. Chromatographic Conditions

All samples were analyzed using an UFLC system (SHIMADZUDGU Corp., Kyoto, Japan). An Agilent ZORBAX SB-C_18_ column (250 mm × 4.6 mm, 5 μm) was used for eluting each sample. The mobile phase consisted of A (acetonitrile with 0.2% formic acid) and B (0.2% aqueous formic acid) with a gradient elution as follows: 1–10 min: 95–80% B; 10–11 min: 80–25% B; 11–14 min: 25–95% B; 14–19 min: 95% B. The flow rate of the mobile phase was 1.0 mL/min, the column temperature was set at 30 °C, and the injection volume was 1 μL.

### 3.6. Mass Spectrometric Conditions

Mass spectrometry detection was performed using an API5500 triple quadrupole mass (AB SCIEX, Framingham, MA, USA) equipped with an electrospray ionization (ESI) source operating in both positive and negative ion modes, and all data were acquired and analyzed by the Analyst 1.6.2 software. The ESI–MS spectra were acquired in the MRM. The parameters in the source were set as follows: GS1 flow: 55 L/min, GS2 flow: 55 L/min, and CUR flow: 40 L/min; gas temperature: 550 °C; pressure of the nebulizer of MS: 4500 V for the positive ion mode and −4500 V for the negative ion mode.

### 3.7. Method Validation

#### 3.7.1. Linearity, LOD, and LOQ

The standard solution containing 43 reference substances was prepared and diluted with water to appropriate concentrations for the construction of calibration curves. Calibration curves were developed by plotting the peak areas versus the corresponding concentrations of each analyte in working standard solution. The LOD and LOQ of 43 constituents were measured at signal-to-noise (S/N) ratios of 3 and 10, respectively.

#### 3.7.2. Precision, Repeatability, Stability, Accuracy, and Matrix Effect

The intra- and inter-day variations were used to evaluate the precision of the established method with the relative standard deviation (RSD) of the peak area as a measure. To confirm the repeatability, six different analytical sample solutions prepared from the same sample were analyzed and expressed by RSD. A stability test was further performed to analyze the variations in the sample solutions at 0, 2, 4, 8, 12, and 24 h, respectively.

A recovery test was used to evaluate the accuracy of the established method. The test was performed in triplicate by adding the corresponding constituents of a known amount at different levels (low, medium, and high, respectively) to 0.5 g of S19. The mixture was extracted and analyzed using the aforementioned method.

Matrix refers to the components of a sample other than the analyte of interest. The matrix can have a considerable effect on the way the analysis is conducted and the quality of the results obtained, for example, ion suppression or enhancement; such effects are called matrix effects. In this study, the matrix effects were evaluated by the slope comparison method. The standard addition method was used by analyzing the sample extracts spiked with appropriate amounts of standards. The slopes of the standard addition calibration curves were then compared with the slopes of the pure water standards. The slope ratio (slope matrix/slope solvent) was calculated to study the matrix effect. When the slope ratio was 1, the matrix does not suppress or enhance the response of the MS; otherwise, ionization suppression or enhancement was indicated [31,32].

### 3.8. Multivariate Statistical Analysis

Multivariate statistical analysis was introduced to analyze the multi-index, multivariate, and other related data that appeared in the study of quality control of traditional Chinese medicine, and the relationship between the laws and data hidden in it can be determined to achieve the effective quality of traditional Chinese medicine evaluation. The combination of the UFLC-QTRAP-MS/MS along with the multivariate statistical analysis details of the samples proved able to identify and select the important markers in samples, even at low concentration levels [33]. PCA is a statistical procedure that uses an orthogonal transformation to convert a set of observations of possibly correlated variables into a set of values of linearly uncorrelated variables called principal components. The normalized data of 41 samples of AVF and PHF tested by UFLC-QTRAP-MS/MS were subjected to PCA to evaluate the differences in chemical composition. Samples were classified on the basis of the content of 43 target constituents using SIMCA-P 13.0 software.

GRA, a multivariate statistical analysis method, was introduced to evaluate the quality of the samples of AVF and PHF based on the concentrations of their bioactive constituents using Microsoft Excel. Through the establishment of sample dataset and normalization treatment of raw data, the optimal and the worst reference sequences were conducted. Then, correlation coefficient and correlation degree were calculated, followed by the relative correlation degree (*r_i_*).

## 4. Conclusions

In this research, an effective and sensitive method was developed and validated for quality evaluation of AVF based on the simultaneous determination of multiple bioactive constituents combined with multivariate statistical analysis. The optimized UFLC-QTRAP-MS/MS technology was successfully applied to quantify 43 bioactive constituents including 15 flavonoids, 6 organic acids, 13 amino acids, and 9 nucleosides in 41 Luobumaye samples (AVF and PHF) from different habitats and commercial herbs. Furthermore, according to the contents of these 43 constituents, PCA was performed to classify and distinguish between AVF and its adulterant PHF, and GRA implied that the relationship of the two species was applied to evaluate the quality of the samples. All of the results demonstrated that PHF was not as good as AVF from the quality point of view, and perhaps it was not the ideal substitute for AVF. In general, the proposed method was quite useful for the overall assessment of the quality of AVF, and this study may provide the foundation and support for normalization and standardization of AVF.

## Figures and Tables

**Table 1:** Precursor/product ion pairs and parameters for MRM (multiple reaction monitoring) of the target compounds.

No.	Constituents	*t*_R_ (min)	[M + H]^+^ *m/z*	[M − H]^−^ *m/z*	Precursor Ion	Product Ion	FV (V)	CE (eV)
1	Glutamic acid	2.02	147.13	-	147.08	83.92	83	14
2	Histidine	2.05	155	-	156.08	110.03	95	16
3	Arginine	2.06	174.2	-	175.12	70.02	88	18
4	Asparagine	2.15	133.06	-	133.06	73.87	65	14
5	Serine	2.18	105.09	-	106.05	59.99	67	8
6	Lysine	2.19	146.19	-	147.11	83.91	66	14
7	Glutamine	2.23	147.08	-	148.06	83.91	58	14
8	Cysteine	2.29	122.15	-	122.03	75.93	85	17
9	Cytidine	2.41	243.22	-	244.09	112	61	10
10	Guanine	2.51	151.12	-	152	135	62	15
11	2′-deoxycytidine	2.57	227.3	-	228.2	112.05	76	13
12	Uracil	2.96	112.09	-	113.04	70	103	13
13	Hypoxanthine	3.2	136.11	-	137.05	137.05	51	24
14	Tyrosine	3.81	182.1	-	182.16	136.08	46	17
15	Isoleucine	3.99	131.18	-	132.1	86.05	98	10
16	Guanosine	4.1	283.24	-	284.3	152	62	15
17	Inosine	4.17	268.23	-	269	137.07	46	15
18	2′-deoxyguanosine	4.36	267.2	-	268.1	152.1	61	15
19	Fumaric acid	4.51	-	115	114.8	70.8	−50	−10
20	Leucine	4.6	131.18	-	132.1	86.05	98	10
21	Gallic acid	5.28	-	169.01	168.9	125	−120	−18
22	Thymidine	6.05	242.23	-	243.1	127.07	61	13
23	Phenylalanine	6.47	165.19	-	166.1	120.05	56	14
24	Neochlorogenic acid	8.28	-	353.09	305.01	125	−95	−20
25	Tryptophan	9	205.09	-	205.03	188.1	61	13
26	Epigallocatechin	9.18	-	305.07	305.1	125	−55	−25
27	Chlorogenic acid	10.14	-	353.09	305.01	125	−95	−20
28	Catechin	10.26	-	289.07	289	244.8	−135	−20
29	Cryptochlorogenic acid	10.44	-	353.09	305.01	125	−95	−20
30	Caffeic acid	11.8	-	179.03	178.97	134.6	−125	−20
31	Epicatechin	11.84	-	289.07	289	244.8	−135	−20
32	Quercetin-3-O-sophoroside	12.6	627.17	-	626.9	303	111	23
33	Quercitrin	12.99	-	447.09	447	301	−165	−30
34	Avicularin	13	435.09	-	435	303	51	15
35	Astragalin	13.01	-	447.09	447.1	283.9	−100	−36
36	Trifolin	13.02	449.11	-	449	287	46	21
37	Hyperoside	13.04	-	463.09	463.003	299.9	−160	−36
38	Isoquercitrin	13.04	-	463.09	463.015	300.0	−180	−36
39	Kaempferol-3-O-rutinoside	13.06	595.17	-	595	287.2	36	25
40	Rutin	13.09	-	609.15	609.06	300	−245	−46
41	Amentoflavone	13.44	539.1	-	539	377	251	57
42	Apigenin	13.6	-	269.05	268.8	116.9	−129	−40
43	Gallocatechin	13.7	-	305.07	305.1	125	−55	−25

**Table 2:** Regression equation, correlation coefficient, linear range, limit of detection (LOD), limit of quantitation (LOQ), precision, repeatability, stability, recovery, and matrix effect of 43 investigated constituents.

Analytes	Calibration Curves	*r*^2^	Linear Range ng/mL	LOD	LOQ	Precision (% RSD, *n* = 6)		Repeatability (% RSD, *n* = 6)	Stability (% RSD, *n* = 6)	Recovery%						Matrix Effect
						Intra-day	Inter-day			Low		Medium		High		
										Mean	RSD	Mean	RSD	Mean	RSD	
Glutamic acid	Y = 642x – 21,200	0.9999	5.02–5020	0.5	1.67	1.85	2.31	2.45	2.34	98.99	3.95	96.09	3.33	100.79	2.39	1.04
Histidine	Y = 1920x – 31,400	0.9999	3.22–3220	0.32	1.07	2.71	3.45	4.37	3.25	102.23	3.67	101.85	3.8	97.32	2.61	1.06
Arginine	Y = 654x + 20,300	0.9993	1.93–3860	1.93	6.43	2.12	2.56	2.56	2.99	99	2.29	102.26	2.99	98.92	3.45	1.00
Asparagine	Y = 130x + 35,400	0.9992	21.37–21,368	2.14	7.12	1.23	1.13	3.24	4.78	98.41	2.54	102.81	4	97.79	3.04	0.98
Serine	Y = 143x + 15,500	0.9995	5.72–5720	5.72	19.07	0.75	2.39	2.47	1.62	99.27	4.71	96.2	2.27	98.24	3.64	1.03
Lysine	Y = 1800x + 358,000	0.9991	4.58–4580	0.46	1.53	3.55	2.53	2.8	3.41	99.06	4.18	99.08	2.02	104	3.64	1.00
Glutamine	Y = 382x + 36,200	0.9993	12.31–12,308	1.23	4.1	1.24	2.36	3.1	2.47	102.56	2.46	102.43	3.09	96.27	1.82	1.01
Cysteine	Y = 3.04x + 949	0.9999	3.31–3310	0.33	1.1	2.14	2.27	3.35	1.21	103.24	2.3	96.77	3.21	100.42	3.64	1.00
Cytidine	Y = 1760x + 68,000	0.9991	7.00–7004	7	23.35	3.18	4.32	2.23	4.28	98.22	1.23	103.83	4	101.79	3.03	0.99
Guanine	Y = 472x + 43,200	0.9994	2.57–2570	2.57	8.57	3.12	2.15	4.12	2.19	96.45	3.66	99.22	4.22	95.84	1.97	1.01
2′-deoxycytidine	Y = 3870x – 88,500	0.9992	2.38–2380	2.38	7.93	1.93	3.86	2.43	3.66	103.08	3.81	99.77	2.87	97.14	1.84	0.97
Uracil	Y = 113x − 5400	0.9993	1.20–602	1.2	4.01	2.67	1.2	3.05	2.76	99.48	2	97.99	2	99.14	1.9	1.01
Hypoxanthine	Y = 3160x + 8150	0.9994	1.31–654	1.31	4.36	4.6	2.67	3.23	2.12	95.12	2.89	98.22	2.01	94.31	1.71	1.03
Tyrosine	Y = 959x + 51,900	0.9992	3.05–3053	0.31	1.02	4.14	3.79	1.77	3.08	100.09	2.31	98.28	3.2	98.12	2.72	1.02
Isoleucine	Y = 1980x + 52,800	0.9992	7.92–7918	0.79	2.64	4.6	3.88	3	3.09	97.8	3.63	103.2	2.74	96.67	2.62	1.04
Guanosine	Y = 1790x − 71,200	0.9998	1.16–579	1.16	3.86	3.96	3.23	2.91	4.67	97.23	2.95	98.2	2.81	94.1	2.8	1.02
Inosine	Y = 2410x − 39,800	0.9998	4.04–4040	4.04	13.47	2.13	4.3	2.44	2.1	98.55	1.89	95.98	1.98	96.3	2.55	0.95
2′-deoxyguanosine	Y = 2500x – 31,200	0.9991	6.94–6939	6.94	23.13	3.71	1.59	4.11	3.45	102.49	2.11	99.27	2.17	97.26	4.23	1.00
Fumaric acid	Y = 106x + 35,600	0.9998	4.44–8880	4.44	14.8	1.85	1.43	3.2	1.23	102.13	3.99	98.12	4.36	97.9	2.52	0.99
Leucine	Y = 1800x + 358,000	0.9991	5.68–5680	5.68	18.93	2.34	3.41	3.45	3.12	100.01	4.29	100.2	4	94.32	1.83	1.01
Gallic acid	Y = 661x – 69,900	0.9991	1.49–1488	1.49	4.96	2.1	3.41	3.74	1.87	96.98	2.84	101.87	2.54	97.93	1.64	0.96
Thymidine	Y = 708x – 32,500	0.9996	3.50–1750	3.5	11.67	2.83	4.37	2.89	4.15	97.09	4.76	100.9	4.65	102	3.37	1.03
Phenylalanine	Y = 1480x + 120,000	0.9997	3.21–3210	0.32	1.07	2.21	3.42	2.68	4.11	101.32	2.3	100.7	3.71	98.93	2.31	1.00
Neochlorogenic acid	Y = 516x + 104,000	0.9996	63.59–254,359	31.79	105.98	2.23	2.63	1.05	1.41	99.65	1.53	103.21	3.3	101.2	3.65	1.00
Tryptophan	Y = 1600x + 518,000	0.9996	17.18–17,184	1.72	5.73	2.68	2.76	3.07	2.54	98.85	2.85	99.9	3.7	98.99	2.81	1.01
Epigallocatechin	Y = 253x – 12,900	0.9993	48.60–48,600	24.3	81	1.74	1.53	1.45	2.47	97.49	1.42	95.61	1.18	97.15	0.86	1.02
Chlorogenic acid	Y = 678x + 650,000	0.9993	45.20–45,200	4.52	15.07	2.88	1.85	2.02	2.31	97.15	1.69	96.21	2.32	96.07	3.96	1.01
Catechin	Y = 142x + 9680	0.9991	6.93–6930	6.93	23.1	1.72	2.39	2.07	3.64	96.24	3.61	102.08	2.35	100.22	3.11	0.96
Cryptochlorogenic acid	Y = 240x – 102,000	0.9997	23.80–95,200	2.38	7.93	2.63	1.33	3.57	2.32	96.91	2.39	99.86	3.45	96.29	2.72	1.04
Caffeic acid	Y = 824x + 184,000	0.9997	16.25–16,250	1.63	5.42	2.47	1.57	2.91	0.95	100.08	4.6	104.3	2.99	101.4	2.06	1.03
Epicatechin	Y = 163x + 129,000	0.9996	13.43–26,864	6.72	22.39	2.46	1.67	2.24	1.62	96.01	1.44	103.43	4.01	99.01	3.14	1.02
Quercetin-3-O-sophoroside	Y = 825x – 109,000	0.9995	21.47–85,867	10.73	35.78	1.99	1.27	2.88	2.29	95.46	2.07	97.06	2.69	102.25	1.18	0.98
Quercitrin	Y = 691x – 41,200	0.9995	3.29–3292	3.29	10.97	1.48	2.8	2.49	2.74	97.03	3.46	100.97	4.06	95.65	3.69	1.02
Avicularin	Y = 1220x − 387,000	0.9994	13.86–13,864	1.39	4.62	2.63	2.97	3.24	2.13	97.37	1.74	100.01	1.52	97.94	2.14	1.01
Astragalin	Y = 679x + 1,080,000	0.9993	21.22–21,217	10.61	35.36	2.12	1.74	1.92	2.9	96.64	2.3	98.6	1.3	95.16	2.45	0.97
Trifolin	Y = 5240x + 631,000	0.9992	7.22–14,445	3.61	12.04	1.65	2.55	2.96	2.15	95.48	1.77	102.22	3.42	101.19	4.05	1.01
Hyperoside/Isoquercitrin	Y = 517x + 1,200,000	0.9995	41.14–123,420	41.14	137.13	3.21	3.97	3.90	1.02	98.1	3.9	98.1	4.2	101.2	2.9	1.03
Kaempferol-3-O-rutinoside	Y = 1190x + 220,000	0.9994	5.83–11,668	5.83	19.45	1.2	1.84	1.57	2.77	97.7	1.69	99.89	1.8	100.5	1.07	0.98
Rutin	Y = 0.633x + 363	0.999	4.45–4455	4.45	14.85	1.9	2.14	2.81	2.15	95.99	4.23	100.99	4.46	97.74	3.19	1.03
Amentoflavone	Y = 4.09x − 1690	0.9992	5.58–11,150	5.58	18.58	2.31	2.16	2.12	2.38	95.64	1.53	102.82	2.77	96.2	2.29	1.04
Apigenin	Y = 27.5x − 7930	0.9994	6.92–13,840	6.92	23.07	1.55	2.59	2.75	2.25	96.06	1.53	99.75	0.95	98.05	3	0.98
Gallocatechin	Y = 218x + 80,100	0.9993	18.08–18,080	9.04	30.13	1.58	1.64	3.31	2.55	98.46	1.68	96.34	1.75	99.87	2.49	0.97

**Table 3:** Information of tested samples of AVF and PHF.

Samples	No.	Habits	Batch No.	Origin
AVF	S1	Jilin	150601	Simcare
	S2	Tianjin	17010001	Jiangxi Pharmaceutical Company Limited company
	S3	Tianjin	160214	Songqing Hall pharmacy
	S4	Tianjin	170117003	Jiangxi Pharmaceutical Company Limited company
	S5	Tianjin	160419	Changqingteng pharmacy
	S6	Tianjin	170106005	Jiangxi Pharmaceutical Company Limited company
	S7	Tianjin	17010001	Jiangxi Pharmaceutical Company Limited company
	S8	Jilin	150653	Simcare
	S9	Tianjin	170106009	Jiangxi Pharmaceutical Company Limited company
	S10	Tianjin	170116001	Jiangxi Pharmaceutical Company Limited company
	S11	Tianjin	170108001	Jiangxi Pharmaceutical Company Limited company
	S12	Tianjin		Baixinyuan Pharmacy of Yangzhou
	S13	Xinjiang		Local collection
	S14	Tianjin	170123005	Jiangxi Pharmaceutical Company Limited company
	S15	Tianjin	161125002	Jiangxi Pharmaceutical Company Limited company
	S16	Tianjin	170101	Zhongda Ku of province
	S17	Tianjin	161126025	Jiangxi Pharmaceutical Company Limited company
	S18	Jiangsu		Local herbal medicine market
	S19	Tianjin	170109006	Jiangxi Pharmaceutical Company Limited company
	S20	Jilin	20141201	Caizhilin Pharmacy
	S21	Tianjin	170115001	Jiangxi Pharmaceutical Company Limited company
	S22	Tianjin	170121031	Jiangxi Pharmaceutical Company Limited company
PHF	S23	Xinjiang	170122005	Jiangxi Pharmaceutical Company Limited company
	S24	Tianjin		Local collection
	S25	Xinjiang	170123005	Jiangxi Pharmaceutical Company Limited company
	S26	Xinjiang	170107006	Jiangxi Pharmaceutical Company Limited company
	S27	Xinjiang		Local herbal medicine market
	S28	Xinjiang	170120008	Jiangxi Pharmaceutical Company Limited company
	S29	Xinjiang	0016M60820	Local herbal medicine market
	S30	Xinjiang	161120023	Jiangxi Pharmaceutical Company Limited company
	S31	Xinjiang	170122006	Jiangxi Pharmaceutical Company Limited company
	S32	Xinjiang	170108005	Jiangxi Pharmaceutical Company Limited company
	S33	Xinjiang	170110008	Jiangxi Pharmaceutical Company Limited company
	S34	Xinjiang	170129006	Jiangxi Pharmaceutical Company Limited company
	S35	Hebei		Local herbal medicine market
	S36	Xinjiang	170107006	Jiangxi Pharmaceutical Company Limited company
	S37	Xinjiang	170103006	Jiangxi Pharmaceutical Company Limited company
	S38	Guangxi		Local herbal medicine market
	S39	Nei Menggol		Local herbal medicine market
	S40	Xinjiang	170125001	Jiangxi Pharmaceutical Company Limited company
	S41	Tianjin	161218-5	Local herbal medicine market

**Table 4:** Quality sequencing of the 41 tested samples.

Samples	*r_i_*	Quality-Ranking	Samples	*r_i_*	Quality-Ranking
S1 ^a^	0.5461	11	S23	0.3371	31
S2	0.5873	2	S24	0.3349	33
S3	0.5448	12	S25	0.3628	25
S4	0.5484	10	S26	0.3284	35
S5	0.5504	9	S27	0.3427	30
S6	0.5701	6	S28	0.3262	36
S7	0.5309	14	S29	0.3780	23
S8	0.5532	8	S30	0.3333	34
S9	0.5895	1	S31	0.3535	27
S10	0.4942	20	S32	0.3239	37
S11	0.4915	21	S33	0.3352	32
S12	0.5805	3	S34	0.3542	26
S13	0.5284	16	S35	0.3114	39
S14	0.4653	22	S36	0.3152	38
S15	0.5717	5	S37	0.3670	24
S16	0.5258	17	S38	0.2937	41
S17	0.5774	4	S39	0.3512	28
S18	0.5238	18	S40	0.2944	40
S19	0.5589	7	S41	0.3494	29
S20	0.5300	15			
S21	0.5401	13			
S22	0.5161	19			

^a^ The 41 samples were the same as in Table 3.

## Supplementary Materials

The following are available online. Figure S1, Total ion chromatogram (TIC) of AVF(A) and PHF(B). Figure S2, Representative extract ion chromatograms of multiple reaction monitoring (MRM) chromatograms of the 43 investigated compounds. Figure S3, Chemical structures of 43 compounds analyzed in this study. Table S1, Effects of extraction method, solvent, solvent to sample ratios, and extraction time on the extraction efficiency of investigated. Table S2, Contents of 43 constituents in samples of AVF and PHF.
