# Supplementary material for: Quality Evaluation of Apocyni Veneti Folium from Different Habitats and Commercial Herbs Based on Simultaneous Determination of Multiple Bioactive Constituents Combined with Multivariate Statistical Analysis
Source: Molecules. 2018 Mar 3;23(3):573. doi: 10.3390/molecules23030573 (PMC6017895; doi:10.3390/molecules23030573)
Supplement: Supplementary file 1 [file molecules-23-00573-s001.pdf]

Table S1 Effects of extraction method, solvent, solvent to sample ratios, and extraction time on the extraction efficiency of investigated

Conditions		Peak Area			
		Uracil	Phenylalanine	Neochlorogenic acid	Hyperoside/Isoquercetin
extraction method	ultrasonic extraction	5.19E+03	2.16E+05	5.79E+07	4.77E+07
	refluxing extraction	2.24E+03	1.94E+05	3.21E+07	3.99 E+07
solvent	water	5.18E+03	2.17E+05	5.8E+07	4.77E+07
	25% methanol	3.0E+03	1.5E+04	5.81E+07	4.77E+07
	25% ethanol	3.6E+03	1.46E+04	5.8E+07	4.56E+07
	50% methanol	1.0E+02	1.01E+03	5.82E+07	4.68E+07
	75% methanol	-	2.6E+02	5.86E+07	4.79E+07
	methanol	-	-	5.75E+07	4.58E+07
	50% ethanol	1.3E+02	1.89E+03	5.81E+07	4.57E+07
	75% ethanol	-	3.9E+03	5.82E+07	4.71E+07
	95% ethanol	-	-	5.6E+07	3.33E+07
solvent to sample ratios (v/w)	12.5:1	2.55E+03	9.4E+04	9.2E+06	2.98E+07
	25:1	2.9E+03	1.96E+05	2.7E+07	3.12E+07
	50:1	4.33E+03	2.05E+05	3.59E+07	4.52 E+07
	100:1	5.18E+03	2.15E+05	5.77E+07	4.75E+07
	200:1	5.2E+03	2.13E+05	5.76E+07	4.73E+07
extraction time	15 min	5.5E+02	8.45E+04	4.81E+06	7.32E+06
	30 min,	7.8E+02	1.19E+05	8.99E+06	9.56E+06
	45 min	‘5.22E+03	2.14E+05	5.78E+07	4.76E+07
	60 min	5.22E+03	2.12E+05	5.72E+07	4.74E+07

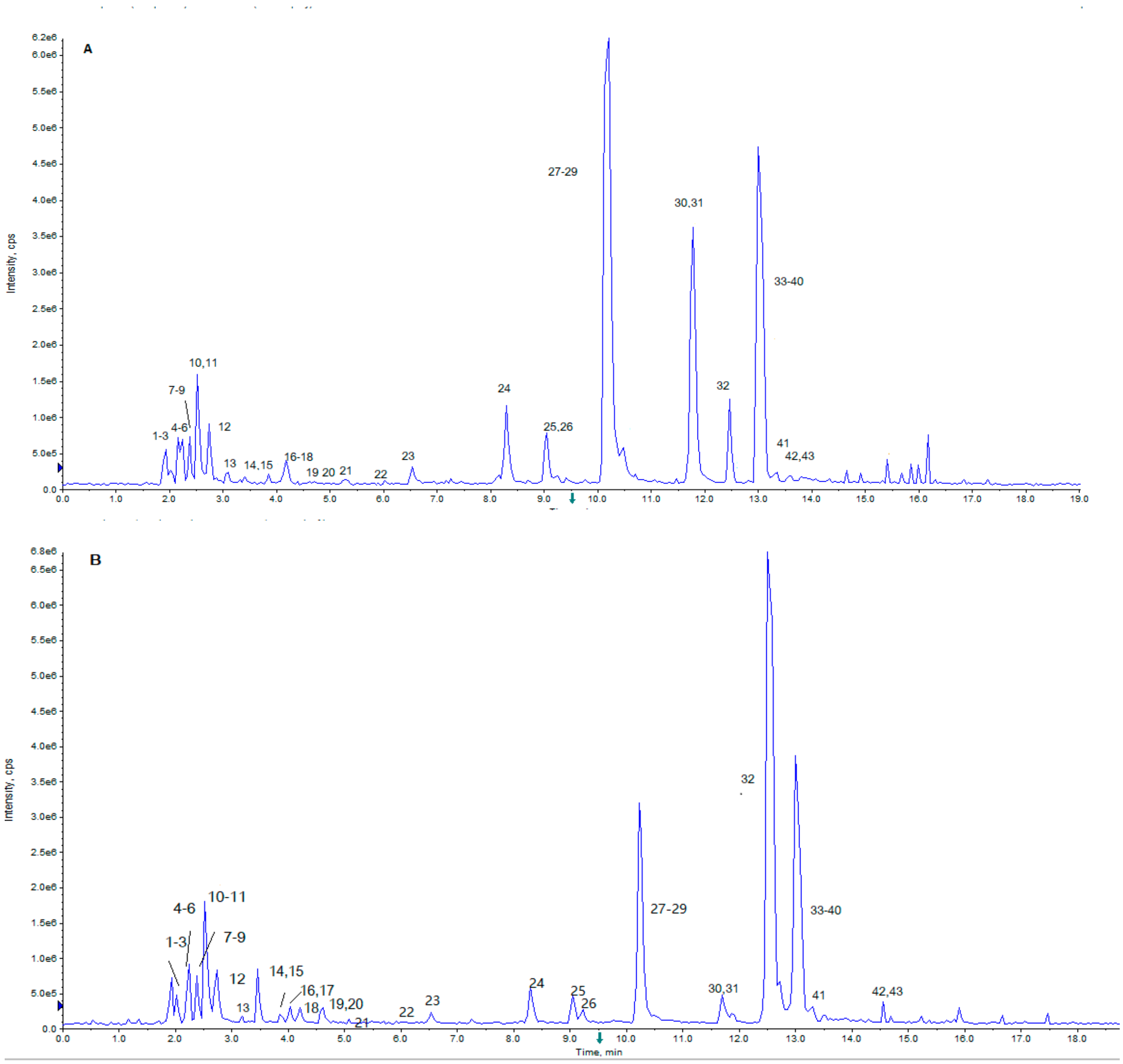

Figure S1 Total ion chromatogram (TIC) of AVF(A) and PHF(B)

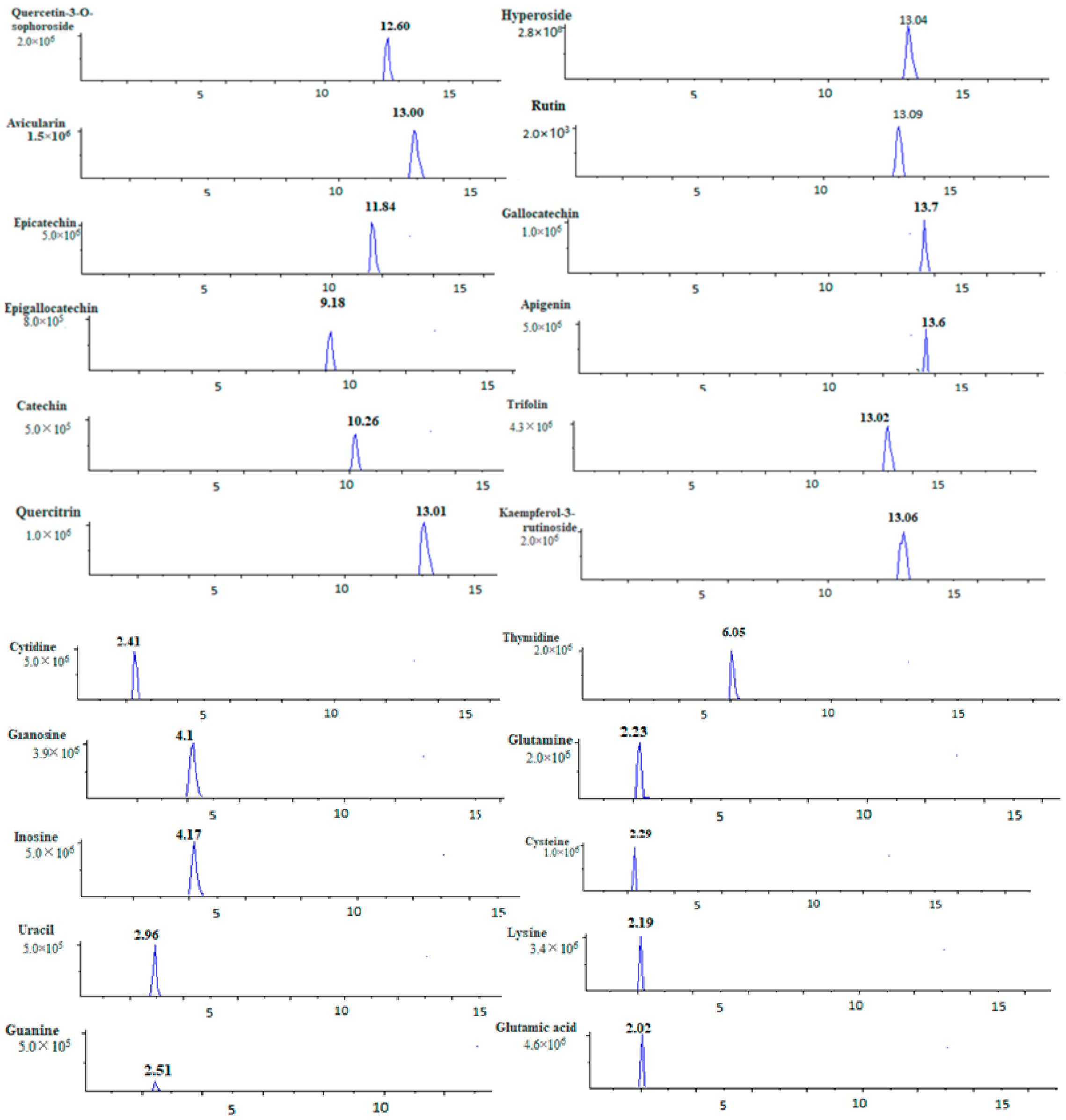

Quercetin-3-O-sophoroside
2.0×10⁵
12.60
Hyperoside
2.8×10⁶
13.04
Avicularin
1.5×10⁶
13.00
Rutin
2.0×10³
13.09
Epicatechin
5.0×10⁵
11.84
Gallocatechin
1.0×10⁶
13.7
Epigallocatechin
8.0×10⁵
9.18
Apigenin
5.0×10⁶
13.6
Catechin
5.0×10⁵
10.26
Trifolin
4.3×10⁶
13.02
Quercitrin
1.0×10⁶
13.01
Kaempferol-3-rutinoside
2.0×10⁶
13.06
Cytidine
5.0×10⁶
2.41
Thymidine
2.0×10⁶
6.05
Gıanosine
3.9×10⁶
4.1
Glutamine
2.0×10⁶
2.23
Inosine
5.0×10⁶
4.17
Cysteine
1.0×10⁶
2.29
Uracil
5.0×10⁵
2.96
Lysine
3.4×10⁶
2.19
Guanine
5.0×10⁵
2.51
Glutamic acid
4.6×10⁶
2.02

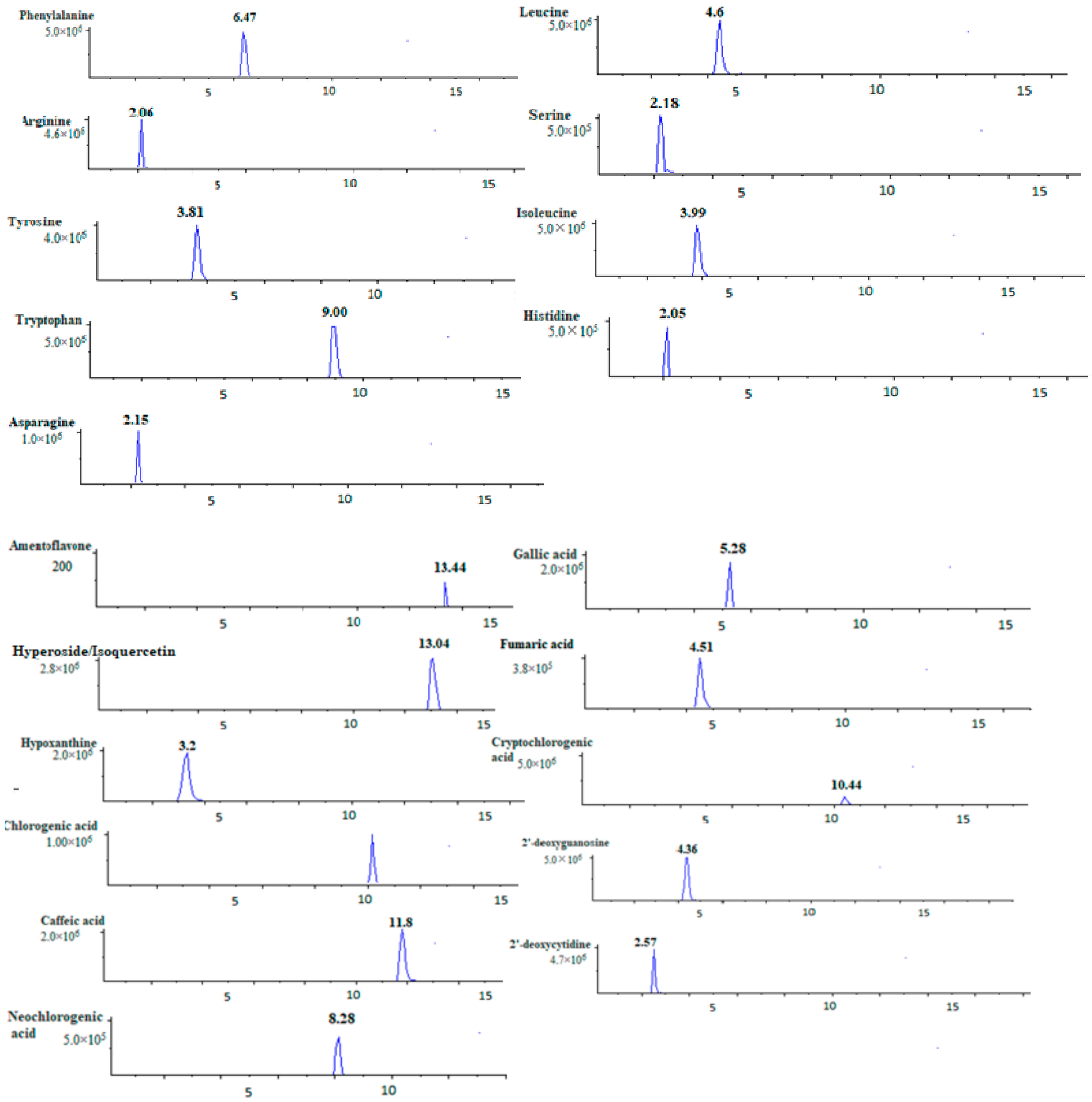

Figure S2 Representative extract ion chromatograms of multiple reaction monitoring (MRM) chromatograms of the 43 investigated compounds.

Table S2 Contents of 43 constituents in samples of AVF and PHF

Sample No.	Contents(μg/g, n=3)									
	**1**[b]	**2**	**3**	**4**	**5**	**6**	**7**	**8**	**9**	**10**
**S1**[a]	133.99	28.53	54.51	952.72	49.98	72.35	458.53	29.91	40.86	24.15
**S2**	118.86	8.38	95.19	568.36	105.54	78.91	383.71	54.75	34.03	28.06
**S3**	79.61	61.09	54.08	550.77	50.57	118.17	297.42	18.78	18.18	30.74
**S4**	102.83	24.68	77.25	430.38	78.75	89.79	452.95	20.47	33.96	11.04
**S5**	72.16	28.41	78.72	812.48	80.24	130.7	323.48	89.32	52.99	27.3
**S6**	141.82	22.74	99.27	947.12	80.53	67.87	409.24	14.38	46.19	32.62
**S7**	91.29	39.68	53.94	446.06	57.05	53.94	393.67	38.9	8.61	16.08
**S8**	109.02	22.5	70.86	446.98	70.37	72.35	385.53	60.46	19.13	2.54
**S9**	109.24	29.8	56.5	585.58	99.68	62.87	409.63	41.92	53.36	14.2
**S10**	78.08	28.26	33.55	366.71	34.46	111.32	299.72	51.88	48.36	11.59
**S11**	74.19	20.88	39.79	345.02	42.53	44	351.12	7.47	45.73	11.43
**S12**	105.97	27.44	57.48	529.63	80.47	74.44	392.34	43.39	59.98	24.39
**S13**	84.88	26.39	54.01	704.73	77.78	38.17	321.19	43.66	46.17	17.49
**S14**	87.66	27.7	52.97	476.78	66.55	99.28	295.83	4.4	35.1	11.38
**S15**	85.57	17.37	73.2	639.18	78.87	142.27	375.77	31.75	46.44	35.31
**S16**	90.39	24.84	50.5	671.58	64.63	52.01	427.19	34.44	16.06	7.07
**S17**	120.16	17.53	44.49	754.72	69.51	196.13	496.52	29.05	21.6	23.44
**S18**	98.56	34.2	47.9	1000.39	87.23	73.43	350.88	14.24	40.21	14.93
**S19**	114.2	30.09	67.39	422.33	84.88	65.33	421.3	45.17	46.04	11.88
**S20**	104.66	27.37	44.12	830.39	55.52	70.76	255.99	25.16	19.41	6.44
**S21**	114.87	86.39	79.63	497.1	94.6	144.79	356.66	36.2	32.05	13.42
**S22**	103.55	30.44	76.58	500.87	103.06	67.42	316.9	42.14	33.33	10.79

**S23**	40.72	26.11	22.76	31.61	33.41	60.02	297.12	5.9	17.76	15.36
**S24**	48.6	19.74	19.36	39.69	26.18	42.91	309.48	16.95	17.27	9.98
**S25**	51.68	18.6	29.49	52.33	29.72	66.63	274.46	34.21	28.67	17.35
**S26**	41.13	28.21	34.45	11.63	20.62	64.9	250.6	27.91	12.08	4.11
**S27**	42.03	24.16	25	42.87	22.48	59.75	267.3	35.27	21.49	7.23
**S28**	39.2	15.13	23.92	26.22	32.71	60.81	281.58	9.91	15.74	8.94
**S29**	19.69	21.02	11.12	15.26	17.5	56.12	164.8	28.37	3.79	6.48
**S30**	48.12	16.8	47.07	44.09	36.49	51.39	266.6	1.82	29.29	5.8
**S31**	47.68	26	29.01	39.67	32.76	46.8	330.7	44.61	27.61	8.37
**S32**	42.33	19.53	23.76	45.22	19.46	52.3	241.21	4.47	19.71	5.44
**S33**	35.61	8.25	24.96	43.81	16.18	52.16	305.51	1.5	18.2	7.08
**S34**	46.45	21.3	28.41	59.93	25.9	67.42	366.01	8.13	23.33	15.3
**S35**	19.52	20	29.79	42.67	15.51	57.43	245.99	18.29	5.08	7.11
**S36**	27.99	8.33	42.23	84.27	26.93	62.48	251.84	4.99	10.99	6.49
**S37**	43.18	13.37	32.01	131.06	41.72	43.39	373.33	31.75	28.72	25.01
**S38**	20.19	12.21	21.94	48.31	34.16	57.89	126	9.54	4.07	4.97
**S39**	48.46	19.27	25.17	40.96	32.72	63.56	369.91	17.73	19.76	11.57
**S40**	51.5	21.85	48.54	119.39	40.33	82.54	286.55	30.58	32.91	26.63
**S41**	37.65	10.97	13.7	38.36	22.79	60.64	245.1	14.81	20.27	40.83

	**11**	**12**	**13**	**14**	**15**	**16**	**17**	**18**	**19**	**20**	**21**
**S1**	1.42	7.59	3.35	120.75	92.9	0.92	10.14	3.89	59.91	66.23	13.33
**S2**	1.89	8.38	4.46	119.35	78.6	1	60.17	4.35	13.22	138.59	17.68
**S3**	1.57	5.41	7.91	69.1	56.96	1.13	34.45	4.11	135.79	74.1	16.62
**S4**	2.25	6.82	3.19	115.37	119.19	1.12	63.7	4.15	16.46	105.34	11.92

**S5**	1.83	4.57	1.6	103.96	126.42	1.18	70.15	4.1	14.7	114.55	18.65
**S6**	1.93	3.24	2.5	122.57	93.79	1.16	64.83	4.58	48.62	130.67	15.88
**S7**	1.33	6.07	5.39	114.63	115.7	0.8	38.07	5.05	32.2	105.81	13.15
**S8**	1.54	9.91	6.24	107.04	131.09	0.89	56.49	4.52	41.62	108.03	15.38
**S9**	2.15	15.96	3.45	121.97	91	1.26	42.09	6.6	16.95	104.7	20.06
**S10**	1.32	9.02	6.9	86.14	58.12	0.78	22.67	3.44	37.36	66.49	10.76
**S11**	1.26	8.38	4.57	84.86	133.16	0.81	24.85	3.84	30.16	67.58	10.04
**S12**	2.05	14.99	3.12	109.46	63.73	1.29	62.98	5.55	16.44	103.7	19.14
**S13**	1.61	6.26	4.04	129.78	98.52	0.69	62.76	3.52	24.53	54.84	19.06
**S14**	1.25	9.03	3.63	14.65	87.81	0.76	21.16	3.86	14.72	92.42	8.18
**S15**	1.91	7.99	1.76	106.7	90.59	1.17	63.4	4.26	56.08	134.54	16.95
**S16**	1.47	12.62	6.82	109.57	86.48	0.79	57.06	4.01	73.52	91.4	28.16
**S17**	1.53	8.99	3.31	87.39	112.38	0.98	53.13	5.02	46.47	152.43	11.64
**S18**	1.54	4.27	0.65	70.96	102.99	0.85	44.99	3.67	17.62	119.75	11.86
**S19**	1.85	6.33	3.24	113.68	57.18	1.18	70.99	4.14	12.92	109.05	11.36
**S20**	1.31	4.4	8.16	81.56	117.77	0.92	39.95	5.55	36.05	123.33	15.14
**S21**	1.4	3.73	2.3	114.38	61.51	0.83	43.24	3.48	14.75	143.34	10.46
**S22**	1.4	4.59	1.51	103.55	106.06	0.77	47.39	2.99	11.98	102.1	10.75
**S23**	1.37	5	6.8	29.66	23.27	0.73	33.91	1.39	18.91	31.31	10.75
**S24**	1.45	4.56	1.3	29.02	45.96	0.76	34.17	1.52	16.9	33.79	12.71
**S25**	1.77	6.61	0.3	7.16	31.56	1.02	54.65	1.61	31.08	45.5	11.34
**S26**	1.24	4.01	0.72	23.71	40.81	0.69	28.41	1.65	14.48	23.06	11.73
**S27**	1.38	4.32	1.07	28.72	39.13	0.77	30.61	1.36	21.91	26.68	12.37
**S28**	1.31	4.48	0.44	24.73	22.94	0.74	27.8	1.2	21.46	29.54	9.97
**S29**	1.22	4.49	3.04	10	36.25	0.7	12.3	1.07	10.41	9.95	7.6
**S30**	1.77	5.61	0.8	17.06	45.96	0.94	46.85	1.44	22.05	23.44	9.76

**S31**	1.5	4.52	3.86	32.71	29.06	0.78	39.26	1.4	14.2	31.87	11.7
**S32**	1.41	4.39	6.02	10.57	27.41	0.74	23.96	1.11	19.12	26.84	8.42
**S33**	1.39	5.48	4.64	16.29	8.56	0.76	35.61	1.33	16.71	42.16	11.55
**S34**	1.49	4.8	1.7	31.52	25.4	0.83	47.04	1.51	25.52	40.56	11.72
**S35**	1.29	4.17	5.4	24.87	32.28	0.72	29.95	1.14	17.11	3.75	9.76
**S36**	1.29	6.73	3.54	18.55	14.48	0.7	22.33	1.21	9.44	23.93	9.69
**S37**	1.47	3.26	0.94	36.55	19.83	0.85	48.04	1.89	19.37	35.98	12.69
**S38**	1.2	3.89	0.67	9.68	43.33	0.68	10.51	1.09	17.03	11.34	7.54
**S39**	1.46	5.51	6.57	36.35	20.58	0.77	50.15	1.33	11.17	36.69	11.42
**S40**	1.52	6.07	6.44	31.77	9.75	0.83	50.41	1.64	19.52	48.23	10.07
**S41**	1.41	4.17	7.23	27.85	41.47	0.77	41.79	1.6	10.06	25.12	9.85

	**22**	**23**	**24**	**25**	**26**	**27**	**28**	**29**	**30**	**31**	**32**
**S1**	2.78	81.52	8549.69	249.13	595.78	851.32	23.12	1069.48	61.27	397.94	4626.73
**S2**	3.22	86.31	7457.1	284.57	586.23	1034.82	18.89	1293.66	166.24	353.13	3938.61
**S3**	3.18	41.76	6336.87	130.18	649.95	1020.1	15.98	721.34	255.29	285.2	4738.79
**S4**	3.08	57.18	8792.14	169.54	688.23	892.86	27.59	791.32	98.58	444.09	3616.06
**S5**	3.45	59.04	6820.08	154.42	374.33	961.51	9.61	750.67	173.33	169.29	4491.89
**S6**	3.34	87.12	7506.75	226.9	424.96	963.67	9.79	683.53	132.77	182.34	4252.83
**S7**	3.03	41.18	8423.24	162.86	741.08	931.54	27.5	659.45	57.34	462.79	5444
**S8**	3.1	50.05	8251.07	160.56	635.72	932.28	24.53	1279.11	76.75	406.74	4947.47
**S9**	3.9	66.96	6343.14	205.4	545.3	956.5	38.38	985.87	194.66	405.65	4337.98
**S10**	2.6	40.7	7597.55	125.43	602.83	843.58	22.35	1405.54	66.76	368.06	4715.22
**S11**	2.77	35.26	7407.86	129.57	563.82	913.96	23.16	1238.5	63.01	363.55	5177.25
**S12**	3.6	76.97	6903.9	131.95	597.24	994.94	29.66	1395.66	195.44	435.95	4740.48

**S13**	2.62	57.11	8160.77	186.26	518.76	828.58	23.94	989.64	80.4	511.25	5517.7
**S14**	2.61	23.77	7763.02	150.75	580.41	970.05	16.89	990.23	84.32	276.64	5207.68
**S15**	3.45	61.34	7697.59	156.7	530.18	1050.86	10.09	653.84	297.32	186.26	5080.42
**S16**	3.63	53.02	7116.41	149.97	416.5	942.57	8.99	581.59	231.6	169.53	3554.85
**S17**	2.77	83.42	6914.26	210.03	627.42	996.36	19.18	1217.37	200.46	306.66	5993.25
**S18**	2.9	72.94	8159.54	183.82	883.4	908.07	21.17	995.79	125.1	328.01	4963.84
**S19**	3.11	55.04	9150.89	161.52	717.78	912.21	29.46	983.79	104.39	469.96	4016.45
**S20**	2.87	72.23	6842.24	221.6	611.93	1073.26	15.45	1449.27	165.36	292.58	4347.17
**S21**	2.77	51.16	8810.81	184.36	695.94	907.34	26.24	974.33	60.1	453.02	4299.62
**S22**	2.74	53.94	8828.1	155.56	692.02	948.44	26.92	867.2	54.33	437.68	3586.51
**S23**	2.58	24.86	2200.05	64.03	102.07	179.14	13.01	247.2	19.11	217.25	12697.6
**S24**	2.7	23.28	2326.58	75.63	116.38	202.82	12.23	270.9	19.29	212.76	11267.62
**S25**	2.82	86.52	2511.77	48.21	105.81	184.17	18.73	192.25	27.99	302.97	10651.39
**S26**	2.44	23.86	2648.76	51.42	111.45	196.35	25.13	205.7	20.33	344.62	11338.02
**S27**	2.78	18.19	2344.34	56.6	154.35	228.09	21.03	247.57	22.66	276.73	12545.09
**S28**	2.55	16.92	1917.42	58.77	122.73	206.6	15.37	272.51	13.87	224.86	12745.06
**S29**	2.52	10.2	1715.48	14.18	96.94	205.85	6.11	221.23	21.91	101.53	12416
**S30**	2.63	13.48	2281.78	28.57	101.27	180.88	7.78	285.59	30.32	127.96	11454.88
**S31**	2.56	22.78	2149.89	75.4	147.2	187.19	19.07	185.4	30.18	270.9	12411.31
**S32**	2.69	19.21	2195.68	40.19	139.12	201.12	16.74	295.84	25.59	201.04	12465.27
**S33**	2.59	22.94	2316.2	83.03	126.61	217.73	17.97	353.2	19.45	254.6	12335.36
**S34**	2.77	23.22	2165.03	95.78	162.06	222.89	18.68	383.11	28.91	269.51	12009.6
**S35**	2.64	13.9	1640.11	34.17	121.93	189.52	11.59	294.15	19.44	90.91	11054.4
**S36**	2.49	20.44	1304.08	76.52	71.22	191.4	10.24	149.73	41.52	166.44	12288
**S37**	2.67	35.82	2489.04	156.12	134.72	190.76	21.63	214.3	17.94	397.7	12467.2
**S38**	2.82	11.48	1683.34	50.11	78.94	176.17	4.38	344.86	24.32	83.19	12189.12

**S39**	2.53	22.89	2381.99	91.36	136.54	226.81	21.05	185.89	26.13	262.99	12262.96
**S40**	3.07	29.69	2398.26	146.39	150.18	203.49	25.06	160.48	23.07	393.31	12725.44
**S41**	2.68	20.11	1833.6	70.25	123.09	202.55	17.79	295.25	20.3	230.11	10784

	**33**	**34**	**35**	**36**	**37/38**	**39**	**40**	**41**	**42**	**43**
**S1**	34.2	20.35	260.53	354.39	4343.24	326.06	101.76	298.02	528.71	249.84
**S2**	35.51	23.69	374.4	395.98	5118.49	198.86	96.65	305.54	521.29	308.67
**S3**	35.41	23.01	288.89	361.64	3717.2	168.13	70.7	326.43	504.7	338.61
**S4**	37	24.04	259.76	358.91	4462.39	117.28	53.32	285.71	466.13	339.75
**S5**	39.57	21.25	258.78	399.46	3905.44	144.1	58.26	307.69	532.9	151.53
**S6**	49.84	23.65	385.12	393.7	4708.7	140.78	67.73	308.38	534.85	177.34
**S7**	39.87	24.94	296.1	443.38	4672.31	93.06	66.4	298.09	504.9	378.98
**S8**	39.49	23.5	307.71	432.32	4200.07	119.94	94.96	298.33	468.58	311.33
**S9**	35.6	20.14	327.6	439.1	4916.24	105.62	70.08	306.08	476.52	316.12
**S10**	37.44	22.92	263.13	358.34	4817.14	106.87	58.58	269.94	482.13	326.14
**S11**	41.87	22.71	308.96	433.96	4735.51	101.68	62	292.46	471.22	285.78
**S12**	32.18	18.53	301.99	409.97	4661.5	110.57	67.64	318.38	511.81	272.09
**S13**	40.74	21.01	288.08	364.86	4493.28	178.22	83.2	325.95	494.32	210.62
**S14**	32.09	18.74	272.18	325.3	4140.97	63.54	62.96	310.42	451.26	326.29
**S15**	63.5	21.46	311.2	353.26	4630.93	157.42	69	336.27	504.25	225.98
**S16**	38.94	21.46	355.84	358.77	4290.21	141.82	60	301.62	518.2	159.57
**S17**	36.38	24.86	337.24	346.86	4380.54	334.19	58.76	318.83	516.22	292.22
**S18**	34.93	23.37	259.65	357.49	4381.05	102.71	43.9	290.58	477.82	384.78
**S19**	40.04	25.02	316.8	438.52	4842.8	119.94	47.52	291.9	537.28	348.83
**S20**	33.96	20.77	284.28	422.59	4378.35	141.3	69.1	292.24	475	309.35

**S21**	38.38	23.62	292.82	376.55	4178.64	123.5	75.16	290.34	461.46	329.34
**S22**	39.3	23.08	267.36	445.09	4333.95	124.22	46.96	303.5	442.92	360.62
**S23**	38.67	17.91	129.71	34.38	1736.15	60.89	29.74	7.17	11.61	220.09
**S24**	41.62	19.08	130.95	42.37	2729.48	39.12	23.61	8.11	12.63	211.51
**S25**	37.99	18.66	144.15	74.75	1965.26	39.97	45.88	7.37	12.55	146.84
**S26**	38.19	17.81	125.06	55.91	2094.1	221.64	30.35	7.85	12	225.64
**S27**	41.93	19.65	127.7	46.98	1935.15	58.35	45.56	9.12	12.67	306.95
**S28**	39.86	18.31	195.58	40.15	2232.98	32.78	37.75	8.26	12.45	239.17
**S29**	35.31	17.43	116.48	18.98	2114.87	47.62	37.62	8.23	12.17	89.12
**S30**	44.14	19.31	138.7	48.92	1914.97	57.14	42.86	7.54	13.1	138.44
**S31**	43.11	19.12	174.54	47.28	2267.92	47.7	40.84	7.49	12.75	271.25
**S32**	37.34	17.96	121.34	29.06	2170.87	3.47	41.98	8.05	12.69	201.81
**S33**	42.85	19.76	179.73	47.48	1914.61	57.06	39.7	8.71	13.25	268.43
**S34**	43.99	20.12	136.6	47.64	1953.21	68.28	26.03	8.92	14.64	298.23
**S35**	37.7	18.25	114.6	20.11	2638.29	75.3	41.18	7.58	12.4	81.11
**S36**	37.44	17.63	225.18	34.95	2301.11	2.71	31.54	7.66	11.61	150.3
**S37**	44.02	20.36	237.33	68.05	1875.51	80.27	33.6	7.63	15.47	229.39
**S38**	34.05	16.62	157.9	26.94	2031.66	2.82	26.41	7.05	11.94	330.38
**S39**	40.07	18.44	136.99	45.66	1932.74	55.34	21.4	9.07	13.25	280.37
**S40**	45.89	19.26	128.65	57.97	2617.93	2.84	20.07	8.14	13.19	260.24
**S41**	41.84	18.45	216.86	40.06	2657.21	46.42	21.4	8.1	11.9	187.66

[a] The 41 samples were the same as in Table 3; [b] The 43 analytes was the same as in Table 1.

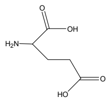

1. Glutamic acid

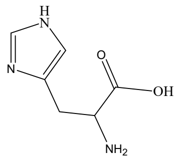

2. Histidine

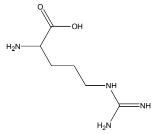

3. Arginine

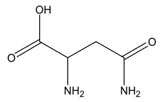

4. Asparagine

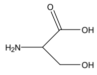

5. Serine

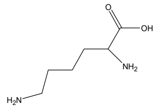

6. Lysine

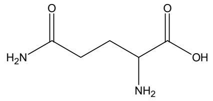

7. Glutamine

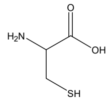

8. Cysteine

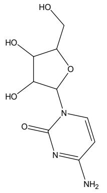

9. Cytidine

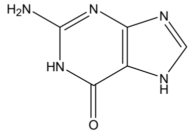

10. Guanine

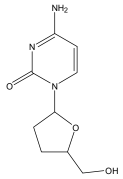

11. 2' -deoxycytidine

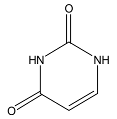

12. Uracil

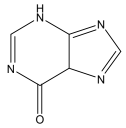

13. Hypoxanthine

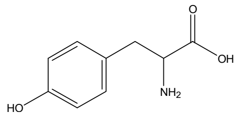

14. Tyrosine

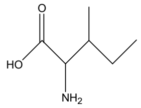

15. Isoleucine

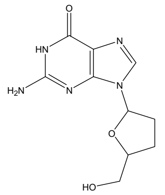

16. Guanosine

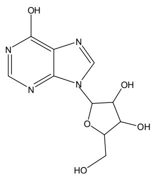

17. Inosine

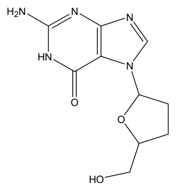

18. 2' -deoxyguanosine

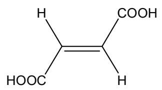

19. Fumaric acid

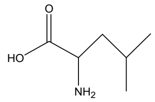

20. Leucine

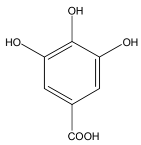

21 Gallic acid

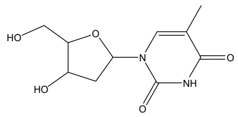

22. Thymidine

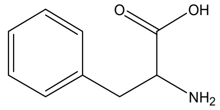

23. Phenylalanine

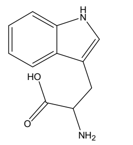

25. Tryptophan

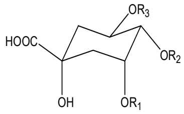

24. Neochlorogenic acid:R_1=R_2=H,R_3=caffeoyl

27. Chlorogenic acid: R_1=caffeoyl, R_2=R_3=H

29. Cryptochlorogenic acid:R_1=R_3=H,R_2=caffeoyl

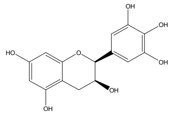

26. Epigallocatechol

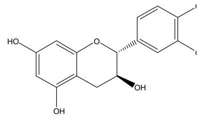

28. Catechin

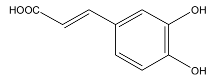

30. Caffeic acid

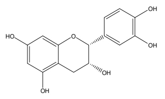

31. Epicatechin

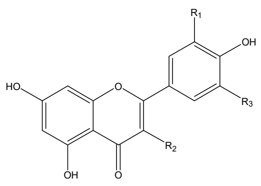

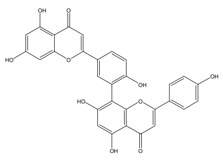

41. Amentoflavone

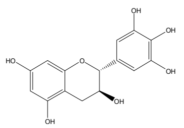

43.Gallocatechin

32. Quercetin-3-O-sophoroside: R_1=OH, R_2=O-sophorose,R_3=H

33. Quercetin: R_1=H, R_2=O-arabinose,R_3=OH

34. Avicularin: R_1=OH,R_2=O-arabinose,R_3=H

35. Astragalin: R_1=H, R_2=O-glucose,R_3=H

36. Trifolin: R_1=H, R2=O-galactose,R_3=H

37. Hyperoside: R_1=OH, R_2=O-galactose,R_3=H

38. Isoquercetin: R_1=OH, R_2=O-glucose,R_3=H

39. Kaempferol-3-rutinoside: R_1=H, R_2=O-galactose,R_3=H

40. Rutin: R_1=OH, R_2=O-rutinose,R_3=H

42. Apigenin: R_1=H, R_2=H,R_3=H

Figure S3 Chemical structures of 43 compounds analyzed in this study
